# Linezolid for the Treatment of Urinary Tract Infections Caused by Vancomycin-Resistant Enterococci

**DOI:** 10.3390/pharmacy9040175

**Published:** 2021-10-26

**Authors:** Mary Joyce Wingler, Neel R. Patel, S. Travis King, Jamie L. Wagner, Katie E. Barber, Kayla R. Stover

**Affiliations:** 1 Department of Antimicrobial Stewardship, University of Mississippi Medical Center, 2500 North State Street, Jackson, MS 39216, USA; mwingler@umc.edu; 2 Department of Pharmacy, Baptist Health Care, 1000 W Morena St, Pensacola, FL 32501, USA; neelpatelpharm@gmail.com; 3 Department of Pharmacy, Ochsner Medical Center, 1514 Jefferson Hwy, New Orleans, LA 70121, USA; samuel.king@ochsner.org; 4 Department of Pharmacy Practice, University of Mississippi School of Pharmacy, 2500 North State Street, Jackson, MS 39216, USA; jwagner@umc.edu (J.L.W.); kbarber@umc.edu (K.E.B.)

**Keywords:** infectious diseases, anti-infectives, genitourinary, enterococcus, vancomycin-resistant, linezolid, urinary tract infections

## Abstract

Vancomycin-resistant enterococci (VRE) account for a large proportion of hospital-acquired infections. Determining optimal treatment of VRE urinary tract infections (UTIs) is challenging. The purpose of this study was to determine if a difference in efficacy or safety exists between linezolid and non-linezolid treatments for VRE UTIs. This retrospective cohort evaluated patients admitted between 1 June 2012–30 November 2017 who were treated for VRE UTI. Patients must have had at least one sign, symptom, or laboratory confirmation of UTI to be included. The primary endpoint of this study was difference in clinical cure between linezolid and non-linezolid treatment options. Secondary endpoints included 30-day recurrence, 30-day infection-related readmission, inpatient mortality, infection-related hospital length of stay (LOS), and time to appropriate therapy. A total of 45 patients (33 linezolid and 12 non-linezolid) were included. Clinical cure occurred in 71.4% linezolid and 58.3% non-linezolid (*p* = 0.476). No patients had a 30-day infection-related readmission or 30-day recurrence. Of the 45 patients, 6 (13.3%) patients died during admission, and 5 of those deaths were in the linezolid group (*p* = 1.000). No significant difference was found for clinical cure between linezolid and non-linezolid treatment options for VRE UTIs.

## 1. Introduction

The most recent National Healthcare Safety Network (NHSN) data from 2011–2014 revealed that *Enterococcus* species were the second leading cause of hospital-acquired infections (HAI), accounting for 14.7% of all reported pathogens [1]. The NHSN data also provide rates of vancomycin-resistance for *Enterococcus faecalis* and *Enterococcus faecium*. Vancomycin-resistance was unsurprisingly higher for *E. faecium* (58.4–86.2%) than *E. faecalis* (3.5–10.1%), and these percentages were similar to previous NHSN reports [1,2,3]. Though rates of vancomycin-resistance have not dramatically increased in recent years, these data do provide evidence of sustained high levels of vancomycin-resistant enterococci (VRE) infections.

Specifically, there have been several studies describing the role of *Enterococcus* spp. and VRE in urinary tract infections (UTIs) [4,5,6,7,8]. In 2020, Gajdacs et al. described the increasing relevance of Gram-positive UTIs, specifically noting the *Enterococcus* spp. are the most prevalent identified organism [4]. Although VRE rates in this report were low (0.16%), high-level aminoglycoside resitance in these infections was described. In contrast, several reports describe the concern with increasing resistance, including resistant strains that form biofilms [5,6,7,8]. Because biofilm encapsulates microorganisms, there is decreased penetration of both antimicrobial and host immunity, resulting in therapeutic challenges [9].

With increasing resistance and concern for biofilm development, choosing optimal treatment for VRE UTIs is challenging due to the limited number of available treatment options. For non-invasive infections such as UTIs, more treatment options are available compared with invasive infections [10,11,12,13]. However, additional elements for treatment must also be considered for UTIs specifically, such as complicating host factors and location of infection (upper versus lower urinary tract). Linezolid is commonly discussed as an option for both invasive and non-invasive VRE infections [10,11,12,13]. Linezolid is an appealing option for VRE UTIs due to an oral formulation, twice daily dosing, no dosage adjustments for renal or hepatic impairment, and availability of a generic option [14]. However, drug-drug interactions, adverse drug reactions, and lack of data for treatment of UTIs are potential limitations [14].

Another potential challenge is achieving adequate drug concentration in the urinary tract because linezolid has lower renal excretion than many other antibiotics utilized for VRE UTIs [15]. A study evaluating renal excretion and bactericidal titers in urine (UBT) compared linezolid with ciprofloxacin and found no difference between the two antimicrobials [15]. Another study compared linezolid and daptomycin for VRE UTIs, showing similar rates of therapeutic failure (1/21 versus 0/8, respectively; *p* > 0.05) and no significant adverse effects for either group [16].

Based on the concerns and limited studies evaluating linezolid for VRE UTIs, it is unclear if linezolid is an optimal choice for VRE UTIs. The purpose of this study was to determine if a difference in efficacy or safety exists between linezolid and non-linezolid treatment options for VRE UTIs.

## 2. Materials and Methods

This retrospective cohort study was conducted at an academic medical center and tertiary referral center in the southeastern United States. This study was approved by the institutional review board (#2017-0302) and the requirement to obtain signed consent was waived. All positive VRE urinary isolates were identified with the hospital clinical decision support system, TheraDoc Clinical Surveillance (Salt Lake City, UT, USA). Patients were included if they had at least 1 documented sign, symptom, or laboratory indication of UTI, had a VRE positive urine culture (≥100,000 CFU; sample collected via standard urine culture protocol and organism identified via Vitek^®^2), or received VRE-directed treatment within 72 h of VRE identification. Signs and symptoms included dysuria, frequency, urgency, new or worsening incontinence, suprapubic tenderness, costovertebral angle tenderness, altered mental status, white blood count (WBC) <4000 or >12,000 cells/mm^3^, temperature ≥38.5 C, pyuria with urinary WBC >5 cells/HPF, hematuria) [17]. Only the first isolate per patient per study period was included. Patients were excluded if they had a polymicrobial UTI (≥2 organisms), died or were discharged within 48 h after the initiation of treatment, were on hospice or placed on comfort care during therapy, had multifocal VRE infection not secondary to UTI, or were treated with combination therapy, sequential therapy, or with antimicrobials active against VRE for an unrelated infection.

The primary endpoint of this study was the difference in clinical cure between linezolid and non-linezolid treatment options for VRE UTI. The secondary endpoints included difference in 30-day recurrence, 30-day infection-related readmission, inpatient mortality, infection-related hospital LOS, and time to appropriate therapy.

Clinical cure was defined as resolution of signs and symptoms of UTI and lack of microbiological persistence. Microbiologic persistence was defined as growth of the bacterial pathogen after completion of therapy (eradication was presumed in patients with clinical improvement and no microbiological follow-up). Patients who were discharged on antimicrobial therapy were considered clinically cured if not re-admitted for a VRE UTI within 30 days. Time to appropriate therapy was defined as the time in hours from the first positive VRE urine culture to the initiation of antimicrobials active against VRE. Infection-related hospital length of stay was defined as the number of days from the date of positive urine culture until discharge. Recurrence was defined as the reappearance of UTI signs and symptoms or VRE positive urine culture up to 28 days after the end of treatment. Infection-related readmission was defined as 30-day readmission due to recurrence of VRE UTI.

Data was collected using REDCap [18] and included pertinent demographic information; comorbid conditions; antibiotic allergies; urologic abnormalities including permanent indwelling urinary catheter, ileal conduit, and intermittent catheterization; clinical and laboratory indicators of UTIs; clinical and microbiological response (i.e., defervescence, normalization of WBC, clearance of urine cultures); basic metabolic profile; treatment-related information, including dose, route, frequency, time to appropriate therapy and duration of antimicrobial treatment; and bacterial identification and susceptibility information.

All statistical analysis were performed using SPSS Software, version 24.0 (SPSS, Inc., Chicago, IL, USA). Categorical data was assessed using Chi-square test and Fisher’s exact test, as appropriate. Continuous data was assessed using Mann–Whitney U. A *p* value ≤ 0.05 was considered statistically significant.

## 3. Results

Of the 116 patients who were screened, a total of 45 patients met inclusion criteria. Thirty-three patients were in the linezolid group and 12 in the non-linezolid group. The primary reasons for exclusion included receiving no VRE-targeted antibiotics (59/116 [50.8%]) and having a polymicrobial UTI (24/116 [20.6%]). In the non-linezolid group, the antibiotics utilized were daptomycin (4 patients, 33.3%), aminopenicillins (5 patients, 41.7%), nitrofurantoin (1 patient, 8.3%), piperacillin/tazobactam (1 patient, 8.3%), and tigecycline (1 patient, 8.3%). The median age for all patients included in the study was 50 years of age (IQR, 36.5–65.5 years), and the majority of patients were Black (24 [53.3%]) and female (34 [75.6%]) (Table 1). Most patients (29 [64.4%]) had a temporary indwelling urinary catheter in both the linezolid and non-linezolid groups (23 [69.7%] versus 6 [50%], *p* = 0.296). Three (6.7%) had a permanent indwelling urinary catheter (2 linezolid, 1 non-linezolid).

This correlated with a high number of catheter-associated UTI (CA-UTIs) in both groups (25 [75.8%] versus 7 [58.3%]). Most of the remaining patients in the linezolid and non-linezolid groups had cystitis (7 [21.2%] versus 5 [41.7%]) (Table 2). The majority of patients had an infection with *E. faecium* in the linezolid and non-linezolid groups (33 [100%] versus 8 [66.7%], *p* = 0.003). In the non-linezolid group, the remaining infections were caused by either *E. faecalis* or other *Enterococcus* species. There were no differences in urinary symptoms between groups.

Many *Enterococcus* isolates were resistant to multiple antibiotic classes in this study (Table 3). Tetracyclines displayed the highest resistance rates with 97% in the linezolid group and 91.7% in the non-linezolid group. Aminopenicillins had higher levels of resistance in the linezolid group (100%) versus the non-linezolid group (50%). Similarly, more resistance to nitrofurantoin was seen in the linezolid group (75.8%) compared with the non-linezolid group (33.3%). Only one isolate demonstrated linezolid-resistance, and this patient was in the non-linezolid group (8.3%); no isolates showed daptomycin resistance. Linezolid and daptomycin MICs were also evaluated, and the median MIC for both linezolid and daptomycin was 2 mcg/L.

The primary endpoint of clinical cure was similar between the linezolid and non-linezolid groups (71.4% versus 58.3%, *p* = 0.476) (Table 4). The median time to appropriate therapy in the linezolid and non-linezolid group was 61 (IQR, 46.5–71) hours and 66 (IQR, 35.5–69.75) hours (*p* = 0.909), respectively (Table 3). The median duration of therapy was significantly longer in the linezolid group (9 (IQR, 6–11.5) days versus 5 (IQR, 4–7.5) days (*p* = 0.002)) (Table 3).

Three patients in our study experienced thrombocytopenia during their course of linezolid with a > 50% drop in platelets. Six patients were thrombocytopenic when linezolid was initiated, but none of these patients had a significant drop in platelets during linezolid therapy. No other adverse reactions were specifically tracked or recorded.

Hospital length of stay (31 (IQR, 19–37) days versus 23 (IQR, 11.25–40.5) days, *p* = 0.367) and infection-related length of stay (13 (IQR, 10–24) versus 9 (IQR, 6.25–23.5) days, *p* = 0.395) were numerically longer in the linezolid group, but the differences were not statistically significant (Table 4). There were no infection-related 30-day readmissions in either group, and 30-day mortality was 5 (15.2%) versus 1 (8.3%) in the linezolid and non-linezolid groups, respectively (*p* = 1.000) (Table 4).

## 4. Discussion

*Enterococcus* species are a leading cause of HAIs, including infections of the urinary tract [1,4]. Little information is available regarding the optimal treatment option for patients with VRE UTIs. In this study, no difference in clinical cure was found between linezolid and non-linezolid treatment options for VRE UTIs. Clinical data supporting the use of linezolid for VRE UTIs are sparse, but these data presented here may provide evidence for use in select patients.

One concern with linezolid use for VRE UTIs has been the lower renal excretion. As briefly described previously, Wagenlehner et al. conducted a study evaluating UBT for linezolid compared with ciprofloxacin to determine if linezolid was comparable to a drug often utilized for UTIs [15]. This study was conducted prior to the widespread fluoroquinolone resistance in Gram-positive organisms, and no difference was found in renal excretion or UBTs between the two antibiotics. The authors concluded there was no difference in bactericidal activity in the urine between linezolid and ciprofloxacin, and linezolid 600 mg twice daily would provide bactericidal activity throughout a 24-h time period [15]. In another study, Pontefract et al. compared treatment outcomes of linezolid versus other options (penicillins (13%), nitrofurantoin (12%), daptomycin (8%), tetracyclines (7%), and fosfomycin and quinupristin/dalfopristin (1% each)) [19]. Linezolid was prescribed in 59% of cases, and no differences between groups were seen in rates of recurrence, retreatment, or mortality. Similarly, linezolid had a high rate of clinical success in our study, demonstrating that appropriate levels of linezolid were likely achieved in the urinary tract.

Drug-drug interactions can occur with serotonergic and adrenergic agents due to linezolid being a weak, non-selective, reversible inhibitor of monoamine oxidase A and B. A review of serotonergic interactions with linezolid found the incidence of serotonin syndrome was 0.24% to 4% [20]. Despite the low prevalence, close monitoring is necessary in patients taking serotonergic agents and linezolid if the combination cannot be avoided, even for the short durations utilized for UTIs [20]. Incidence of drug interactions was not evaluated in this study. Future studies should evaluate the significance of drug interactions with linezolid in the treatment of VRE UTIs to determine if this is a major deterrent to its use.

Adverse drug reactions (ADRs) are commonly seen with linezolid, but more serious ADRs are often limited to patients on linezolid for >14 days [14]. Myelosuppression is thought to occur due to suppression of mitochondrial respiration, and is reversible when linezolid is discontinued. However, more recent evidence suggests that linezolid-associated thrombocytopenia (LAT) commonly occurs in patients with shorter durations of therapy [21]. Rabon et al. evaluated LAT in patients receiving >7 days of linezolid. The median duration of therapy was 9 days, and LAT occurred in 35.8% of patients despite the majority of patients (84.3%) receiving <14 days of therapy [21]. Other ADRs related to its mitochondrial mechanism include peripheral neuropathy and optic neuropathy, which are also typically only seen in patients on prolonged courses of therapy [14]. Because the treatment duration for VRE UTIs would not be >14 days, expected ADRs would include GI upset, headache, and insomnia, but thrombocytopenia should be monitored closely based on new evidence [14]. Our study had limited patients with thrombocytopenia in the linezolid group (3/33 [9.1%]), likely correlating with the short courses of therapy.

The treatment of VRE UTIs is simple in patients who do not have drug allergies or the organisms are not multidrug-resistant. However, resistance to other classes is often present with VRE isolates, and evaluation of linezolid is needed to provide alternative treatment recommendations [13,22]. In addition, appropriate use of linezolid should be taken into consideration from an antimicrobial stewardship standpoint. Taimur et al. evaluated the treatment of vancomycin-susceptible and resistant enterococcal UTIs and found a high percent of inadequate treatment [23]. The authors recommended creating clinical education programs and algorithms to guide therapy of enterococcal infections, particularly VRE [23]. An example algorithm has been published by Heintz et al. in order to provide guidance [12]. In this algorithm, linezolid was recommended as an alternative for ampicillin-susceptible and ampicillin-resistant VRE isolates for patients with cystitis. Linezolid was also recommended as an alternative for pyelonephritis or bacteremic UTI for both ampicillin-susceptible and ampicillin-resistant VRE isolates [12]. This algorithm appropriately places linezolid second-line for cystitis and pyelonephritis when other treatment options can be utilized.

In two small, retrospective studies, several treatment options for VRE UTI were evaluated [24,25]. Shah et al. evaluated clinical cure and microbiological eradication in patients receiving ampicillin for complicated VRE UTIs [24]. Despite ampicillin resistance, a high percentage of patients achieved clinical cure (74/84 [88.1%]) and microbiological eradication (50/58 [86%]). Cole et al. performed a study evaluating the use of aminopenicillins compared with non-beta-lactam antibiotics for VRE UTIs, and further stratified results based on ampicillin susceptibility [25]. Overall, no difference was found in clinical cure between the two groups (26/31 [83.9%] versus 22/30 [73.3%], respectively; *p* = 0.363). The antibiotics utilized in the non-beta-lactam group included ciprofloxacin, linezolid, daptomycin, nitrofurantoin, and fosfomycin. When evaluating clinical cure rates for patients who received ampicillin with ampicillin susceptible and ampicillin non-susceptible isolates, there were similar rates of clinical cure (14/17 [82.4%] versus 12/14 [86%], respectively). This study not only provides evidence for the use of aminopenicillins for ampicillin-resistant VRE UTIs, but also for the use of linezolid. The majority of patients in the non-beta-lactam group received linezolid (22/30 [73.3%]), and the clinical cure rate for this group as a whole was very high [25]. Although previously published results suggest that it may be reasonable to utilize aminopenicillins for ampicillin-resistant VRE UTIs, our results did not include a large enough ampicillin sample to appropriately assess response to that agent [24,25]. However, given these combined results, linezolid should be considered as an alternative therapy in patients who experience treatment failures or have penicillin allergies.

Our study is not without limitations. This study evaluated a small patient population, which may diminish our ability to detect a true difference between groups. However, this population was representative of the patients who develop VRE infections at this institution. In addition, this was a single center, retrospective study; therefore, inherent limitations exist, such as confounding factors and selection bias. To account for this, we utilized strict inclusion and exclusion criteria to increase internal validity. Because a small number of included patients had symptoms (9/33 (27%) in the linezolid group and 4/12 (33%) in the non-linezolid group), it is possible patients with asymptomatic bacteriuria were included. In order to mitigate the risk of including patients with colonization only, other signs were evaluated in order to improve our ability to detect patients with true infection. Next, we considered patients without repeat cultures as having achieved microbiological eradication. Although it is possible some patients were considered cured who actually had unresolved microbiological infection, urine cultures are not routinely performed at this institution as a test-of-cure. Therefore, we feel this is a true representation of how patients are handled clinically at this institution. Finally, it is becoming increasingly important to practice stewardship of culturing in order to prevent unnecessary or prolonged treatments [26,27], particularly in patients without symptoms or those who are responding well clinically to treatment. Further incorporation of stewardship of culturing into UTI treatment protocols may help prevent the treatment of these patients in the future.

## 5. Conclusions

Our study represents a real-world evaluation of the use of linezolid for VRE UTI at a tertiary academic medical center. Our results add clinical data to support the use of linezolid for VRE UTIs. Limited clinical failures were seen, and side effects, namely thrombocytopenia, were observed rarely. Resistance to other treatment options such as ampicillin, nitrofurantoin, and tetracyclines increases the need for alternative treatments. Linezolid’s availability as parenteral and oral formulations, low cost, and limited adverse effects when used for short courses makes it an appealing option for VRE UTIs. Larger studies are still needed to confirm these results. In addition, more studies are needed to evaluate optimal choice of therapy in multidrug-resistant enterococcal UTIs and duration of therapy.

## Figures and Tables

**Table 1:** Patient Demographics.

Variable	Total (n = 45)	Linezolid (n = 33)	Other Antibiotics (n = 12)	*p*-Value
Age in years, median (IQR)	50 (36.5–65.5)	49 (36.5–62.5)	55 (37.75–67.5)	0.502
Race, n (%)				0.366
White	20 (44.4)	16 (48.5)	4 (33.3)	0.28
Black	24 (53.3)	16 (48.5)	8 (66.7)	1.000
Hispanic	1 (2.2)	1 (3)	0 (0)	
Gender, n (%)				0.699
Female	34 (75.6)	24 (72.7)	10 (83.3)	
Penicillin allergy, n (%)	4 (8.9)	3 (9.1)	1 (8.3)	1.000
Charlson comorbidity index, median (IQR)	4 (3–5.5)	4 (2.5–5)	5 (3–6)	0.409
SCr, median (IQR)	1.5 (0.75–2.8)	1.7 (0.75–2.8)	0.80 (0.61–3.09)	0.328
Urologic				
abnormalities, n (%)				
None	13 (28.9)	8 (24.2)	5 (41.7)	0.285
Permanent indwell-	3 (6.7)	2 (6.1)	1 (8.3)	1.000
ing urinary catheter				
Temporary indwelling urinary catheter	29 (64.4)	23 (69.7)	6 (50)	0.296

IQR = interquartile range; SCr = serum creatinine.

**Table 2:** VRE Infection Characteristics.

Variable	Total (n = 45)	Linezolid (n = 33)	Other Antibiotics (n = 12)	*p*-Value
Pyuria, n (%)	23 (51.1)	18 (54.5)	5 (41.7)	0.514
Hematuria, n (%)	27 (60)	21 (63.6)	6 (50)	0.499
WBC, in cells/mm^3^ median (IQR)	5500 (325–16,300)	3750 (225–12,775)	10,250 (1975–17,775)	0.302
Temperature in F, median (IQR)	98.5 (97.9–99.4)	98.5 (97.7–99.7)	98.25 (98.10–98.775)	0.752
Documented indication, n (%)				0.346
Cystitis	12 (26.7)	7 (21.2)	5 (41.7)	
Pyelonephritis	1 (2.2)	1 (3)	0	
CAUTI	32 (71.1)	25 (75.8)	7 (58.3)	
Quantitative culture in CFU/mL, median (IQR)	50,000 (32,500–70,000)	60,000 (25,000–75,000)	50,000 (30,500–52,500)	0.178
*Enterococcus* spp., n (%)				
*E. faecalis*	2 (4.4)	0	2 (16.7)	0.067
*E. faecium*	41 (91.1)	33 (100)	8 (66.7)	0.003
Other enterococcal species	2 (4.4)	0	2 (16.7)	0.067
Resistance, n (%)				
Linezolid	1 (2.2)	0	1 (8.3)	0.267
Daptomycin	0	0	0	--
Aminopenicillin	39 (86.7)	33 (100)	6 (50)	<0.001
Nitrofurantoin	29 (64.4)	25 (75.8)	4 (33.3)	0.014
Aminoglycoside	12 (26.7)	7 (21.2)	5 (41.7)	0.254
Tetracycline	43 (95.6)	32 (97)	11 (91.7)	0.467

VRE = vancomycin resistant enterococci; CVA = costovertebral angle; WBC = white blood count; IQR = interquartile range; CAUTI = catheter-associated UTI; CFU = colony forming units

**Table 3:** Treatment Characteristics.

Variable	Total (n = 45)	Linezolid (n = 33)	Other Antibiotics (n = 12)	*p*-Value
VRE Treatment, n (%)				
Linezolid	33 (73.3)	33 (100)	--	
Daptomycin	4 (8.9)	--	4 (33.3)	
Aminopenicillin	5 (11.1)	--	5 (41.7)	
Nitrofurantoin	1 (2.2)	--	1 (8.3)	
Piperacillin-tazobactam	1 (2.2)	--	1 (8.3)	
Tigecycline	1 (2.2)	--	1 (8.3)	
Daptomycin dose in mg/kg, median (IQR)	5 (4–6)	--	5 (4–6)	
Duration of therapy in days, median (IQR)	8 (5–11)	9 (6–11.5)	5 (4–7.5)	0.002
Time to appropriate therapy in hours, median (IQR)	62 (46.5–71)	61 (46.5–71)	66 (35.5–69.75)	0.909

VRE = vancomycin resistant enterococci; IQR = interquartile range.

**Table 4:** Clinical Outcomes.

Variable	Total (n = 45)	Linezolid (n = 33)	Other Antibiotics (n = 12)	*p*-Value
Composite clinical cure, n (%) *	27/40 (67.5)	20/28 (71.4)	7/12 (58.3)	0.476
Microbiological outcomes, n (%)				
Eradication	45 (100)	33 (100)	12 (100)	1.000
Relapse	0	0	0	
Recurrence	0	0	0	
Defervescence, n (%) *	1/1 (100%)	--	1/1 (100%)	--
Resolution of hematuria, n (%) *	20/25 (80%)	16/19 (84%)	4/6 (67%)	0.562
Resolution of pyuria, n (%) *	17/20 (85%)	14/15 (93%)	3/5 (60%)	0.14
Hospital length of stay in days, median (IQR)	30 (15.5–37)	31 (19–37)	23 (11.25–40.5)	0.367
Infection-related length of stay in days, median (IQR)	11 (8–24)	13 (10–24)	9 (6.25–23.5)	0.395
Discharge disposition, n (%)				0.223
Home	21 (47.7)	14 (42.4)	7 (63.6)	0.195
SNF/LTCF	9 (20.5)	5 (15.2)	4 (36.4)	0.558
Rehabilitation	4 (9.1)	4 (12.1)	0	0.309
Hospice	5 (11.4)	5 (15.2)	0	0.309
Death	5 (11.4)	5 (15.2)	0	
30-day disposition, n (%)				
Alive + not readmitted	28 (62.2)	20 (60.6)	8 (66.7)	1
Alive + non-infection readmission	11 (24.4)	8 (24.2)	3 (25)	1
Dead	6 (13.3)	5 (15.2)	1 (8.3)	1
30-day retreatment, n (%)	0	0	0	1

* = In survivors; AMS = altered mental status; IQR = interquartile range; SNF = skilled nursing facility; LTCF = long-term care facility.

## Data Availability

The data presented in this study are available on request from the corresponding author. The data are not publicly available due to data sharing regulations at our institution.
